# Linking Plant Metabolomics with Fungal Functional Dynamics Reveals a Noncanonical S‐R‐C Adaptive Trajectory

**DOI:** 10.1002/advs.74875

**Published:** 2026-03-18

**Authors:** Peilin Chen, John W. Taylor, Cheng Gao

**Affiliations:** ^1^ State Key Laboratory of Microbial Diversity and Innovative Utilization Institute of Microbiology Chinese Academy of Sciences Beijing China; ^2^ College of Life Sciences University of Chinese Academy of Sciences Beijing China; ^3^ Department of Plant and Microbial Biology University of California Berkeley California USA

**Keywords:** holo‐omics, life‐history strategies, metabolome, mRNA‐seq, mycobiome, phyllosphere

## Abstract

Phyllosphere plant‐fungal interactions are crucial for crop health, yet how leaf metabolite dynamics interplay with fungal functional succession remains unclear. Using field‐based holo‐omics, we show that developmental shifts in sorghum leaf metabolomes drive a noncanonical temporal trajectory of fungal life‐history strategies from stress‐tolerator (S), through ruderal (R), to competitor (C). Young leaves deploy antifungal metabolites that select for stress‐tolerators (S‐strategists) with expanded genomes. A transient increase in maltose abundance during flowering promotes the proliferation of fast‐growing ruderal (R‐) strategists. As plants mature, competitive (C‐) strategists rely on secondary metabolism to persist in crowded communities. We further detect asynchrony between taxonomic and functional beta diversities, and a drop in the taxonomic one coincides with upregulated expression of fungal genes encoding secreted carbohydrate‐active enzymes and effectors. Collectively, these findings identify developmental stage‐specific leaf metabolomes as key determinants of phyllosphere fungal adaptation and functioning.

## Graphical abstract

Using field‐based holo‐omics, we demonstrate that developmental shifts in sorghum leaf metabolomes drive a noncanonical fungal succession from stress tolerators (S) through ruderals (R) to competitors (C). Antifungal metabolites in young leaves select for S strategists with expanded genomes, transient maltose pulses during flowering favor fast‐growing R strategists, and secondary metabolites in mature leaves support C strategists in crowded phyllosphere communities.

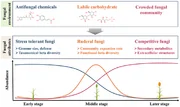

## 1 Introduction

The phyllosphere, a vast microbial habitat covering over 10^8^ km^2^ of leaf surface area on Earth, forms a key interface between plants and the atmosphere and is fundamental to plant fitness, ecosystem stability, and planetary health [1]. This niche harbors taxonomically and functionally diverse microbial assemblages that mediate processes such as nutrient turnover, carbon flux, and pathogen suppression [2, 3, 4, 5]. Within the phyllosphere, fungi (including mutualists, commensals, saprotrophs, and pathogens) exert outsized influence on host physiology and community dynamics because of their metabolic versatility and intimate ecological interactions with plants [6, 7, 8].

Phyllosphere fungal assemblages change over time [9, 10, 11, 12]. For example, our earlier field study of sorghum documented a progressive decline in fungal beta diversity across the growing season concomitant with a shift from stochastically to deterministically structured assembly processes [9]. However, descriptive community trajectories alone do not reveal the underlying adaptive strategies or the functional traits that permit particular taxa to survive or thrive at different developmental stages. A critical unresolved question is whether functional beta diversity exhibits patterns similar to taxonomic beta diversity. Furthermore, based on the established yet controversial beta diversity‐ecosystem functioning framework [13, 14], can we expect the changes of fungal beta diversity to be relevant to ecosystem‐level processes, such as pathogen suppression?

In bacterial ecology, genomic trait‐based metrics (e.g., average genome size, rRNA operon copy number, and codon‐usage proxies of growth potential) have been instrumental in linking community shifts to ecological strategies, such as r‐K selection, the copiotroph‐oligotroph continuum, Grime's Competitor‐Stress‐tolerator‐Ruderal (C‐S‐R) framework, and the recently developed high‐Yield‐resource Acquisition‐Stress‐tolerator (Y‐A‐S) framework [15, 16, 17, 18, 19, 20]. Originally developed for plant ecology, Grime's C‐S‐R framework categorizes species into three primary strategies based on environmental conditions: Competitors (C) dominate in productive, low‐stress habitats through resource competition; Stress‐tolerators (S) persist under resource limitation or abiotic stress; and Ruderals (R) thrive in frequently disturbed environments through rapid growth and reproduction [15]. This framework has proven useful for understanding microbial community dynamics in variable environments, including plant‐associated microbiomes experiencing temporal changes in resource availability and environmental stress [16].

Largely because fungal reads are very low in metagenomic datasets [21], comparable omic trait‐based frameworks for fungi are underdeveloped, although recent work has shown that taxon‐level assignments can be used to estimate average fungal genome size and GC content [22]. However, the biological and ecological significance of genome size in fungi may differ from that in bacteria. In bacteria, genome size correlates tightly with gene number and metabolic versatility [17]. In fungi, genome size variation is driven largely by non‐coding elements (transposable elements, intergenic regions) rather than gene content alone, and fungi exhibit remarkable ploidy plasticity as an adaptive mechanism [23]. Nevertheless, Zhang et al. [22] showed that fungal genome size and nucleotide composition vary systematically along soil fertility gradients, suggesting ecological relevance. Thus, while the mechanisms linking genome size to fungal ecology remain less direct than in bacteria, genome size may still be an ecologically meaningful trait warranting further investigation in trait‐based frameworks. Besides, plant mRNA‐seq datasets frequently contain other eukaryotic (including fungal) transcripts due to shared polyadenylation (poly(A)) tail, and several studies have exploited this “passenger” signal to probe root mycobiome function and plant‐fungal interactions [24, 25, 26]. Despite these advances, integrated omic trait‐based analyses have not yet been systematically applied to the mycobiome, leaving a critical gap regarding phyllosphere fungal adaptation across plant development. Here, we propose a simple, heuristic hypothesis, H_1_, that fungal functional genomic attributes exhibit systematic temporal shifts aligned with plant developmental stages.

Assembly and functioning of the phyllosphere mycobiome are strongly shaped by host traits [2, 27, 28, 29, 30, 31, 32, 33]. A central mechanism by which hosts influence fungal communities is the leaf metabolome: by provisioning nutrients and deploying antimicrobial and signaling compounds, the metabolome both filters colonists and modulates microbial activity [34]. Because plant metabolite profiles change substantially through ontogeny from seedling stages through flowering to maturity, developmental metabolomic shifts are poised to generate corresponding temporal dynamics in mycobiome composition and function. Several studies revealed metabolite‐driven modulation of bacterial phyllosphere communities [34, 35, 36, 37], but comparable mechanistic insights for fungi remain limited. For example, important progress has been made by Edwards, et al. [38], who demonstrated correlations between fungal community composition and overall metabolome dissimilarity and revealed that experimental warming alters this relationship. Building on this foundation, a key frontier for the field is integrating the temporal resolution of specific metabolite dynamics with fungal taxonomic and functional trait responses, which would enable a mechanistic understanding of fungal adaptive strategies in the phyllosphere. Here, we propose another heuristic hypothesis, H_2_, that temporal changes in fungal functional attributes correlate with the dynamics of specific leaf metabolites, revealing underlying life‐history strategy tradeoffs among phyllosphere fungi.

Here, we address these hypotheses by linking fungal functional traits to leaf metabolomes across sorghum development in a semiarid agricultural field (Figure S1). We integrate leaf mRNA‐seq, fungal internal transcribed spacer 2 (ITS2) metabarcoding, and untargeted leaf metabolomics derived from previously published field datasets [39, 40, 41] to infer community‐weighted genomic attributes and to map these traits onto changes in metabolite composition through time (Figure S1). Using this holo‐omics framework, we show that developmental shifts in leaf metabolites drive shifts in fungal functional attributes and life‐history strategies, producing a noncanonical successional trajectory from stress‐tolerators (S), via ruderal (R), to competitors (C). We additionally identify a temporal decoupling between taxonomic and metatranscriptomic beta diversity, and we link the decline in beta diversity in later leaves to increased expression of fungal genes encoding secreted carbohydrate‐active enzymes and effectors.

## 2 Results

### 2.1 Temporal Turnover of Functional Attributes of Phyllosphere Mycobiome

**FIGURE 1:**
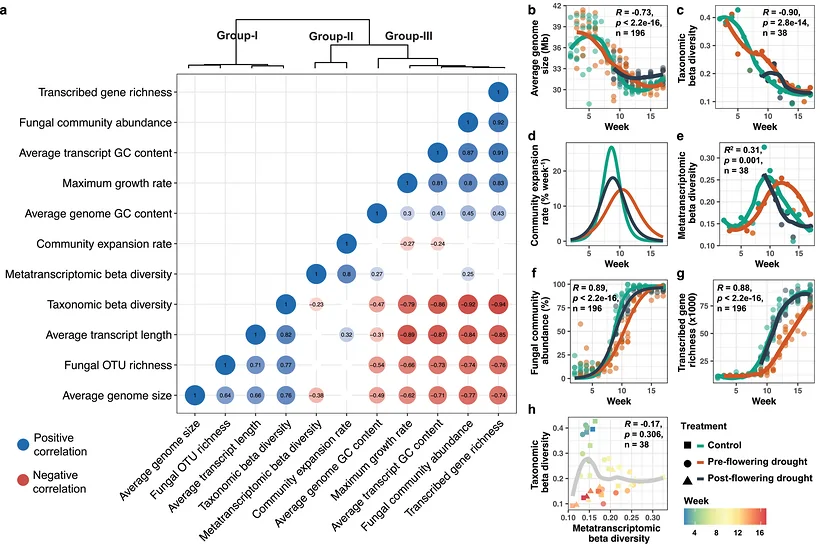
Temporal dynamics and grouping of fungal attributes. (a) Eleven fungal attributes clustered into three groups based on temporal patterns. Group I (decreasing): average genome size, taxonomic beta diversity, average transcript length, and fungal OTU richness. Group II (hump‐shaped): beta diversity of metatranscriptomes and community expansion rate. Group III (increasing): fungal community abundance, transcribed gene richness, maximum growth rate, average genome GC content, and average transcript GC content. Numbers in the heatmap represent Spearman's correlation coefficients (*n* = 196). (b,c) Examples of Group I: (b) average genome size and (c) taxonomic beta diversity decreased over time. The decrease in average genome size was driven by a decline in large‐genome taxa (34.2−89.6 Mb) and a concurrent increase in small‐genome taxa (17.3−33.2 Mb) over time (see Figures S3 and S4). (d,e) Examples of Group II: (d) community expansion rate and (e) beta diversity of metatranscriptomes exhibited hump‐shaped dynamics, increasing from the seedling to the flowering stage and decreasing from the flowering to the grain‐filling stage. (f,g) Examples of Group III: (f) fungal community abundance and (g) transcribed gene richness increased over time. Temporal dynamics of other attributes (average transcript length, fungal OTU richness, maximum growth rate, average genome GC content, and average transcript GC content) were shown in Figure S2. (h) Decoupling between taxonomic beta diversity and metatranscriptomic beta diversity.

To determine whether functional (gene‐expression) dynamics mirror previously reported taxonomic shifts, we compared temporal trajectories of fungal taxonomic (ITS2 metabarcoding) beta diversity with metatranscriptomic (gene‐expression composition) beta diversity (Figure 1). While taxonomic beta diversity declined monotonically over host development (Pearson's R = −0.90, *p* = 2.8×10^−14^) [9], functional (metatranscriptomic) beta diversity followed a pronounced hump‐shaped trajectory across plant developmental stages (quadratic regression: R^2^ = 0.31, *p* = 0.001), demonstrating a decoupling between taxonomic and functional beta diversities (Pearson's R = −0.17, *p* = 0.306) (Figure 1c,e,h).

Because prior work showed that taxonomic beta diversity drops as fungal community abundance increases (proportion of fungal reads in ITS2 metabarcoding) [9], we asked which community attributes track the unique temporal pattern of metatranscriptomic turnover. We therefore assembled a suite of community descriptors from both ITS2 metabarcoding and metatranscriptome datasets. We modelled fungal community abundance (N) over time (t) with a logistic growth curve (logistic model: *p* < 1e^−16^). From that fit, we derived the fungal community expansion rate (g) as the time derivative of abundance, g = dN/dt [42]. The fungal community expansion rate itself showed a hump‐shaped temporal profile (Figure 1d) and was strongly positively associated with metatranscriptomic beta diversity (Spearman 𝑟 = 0.80; Figure 1a,d,e), implicating transient community proliferation as a major correlate of functional overdispersion.

Paralleling these dynamics are concurrent changes of a number of genomic attributes. Over time, we observed a decline in average transcript length and fungal operational taxonomical unit (OTU) richness, together with an increase in average transcript GC content (Figure 1a; Figure S2). Using fungal OTU identities (ITS2 metabarcoding) matched to genome metadata from NCBI, we estimated average genome size and genome GC content (genomic information for the closest reference genomes of the top 25 most abundant fungal OTUs are detailed in Table S1), finding that average genome size decreased significantly from seedling to grain maturation while average genome GC content increased (Figure 1b; Figure S2). Tracking the top 25 most abundant OTUs (average 88.85% of total fungal reads) showed that the average genome‐size decline was driven by increases in the relative abundance of small‐genome taxa (17.3−33.2 Mb; predominantly six Basidiomycota yeasts and two small‐genome Ascomycota taxa) and by decreases in larger‐genome Ascomycota taxa (34.2−89.6 Mb) (Figures S3 and S4). Functional guild assignments based on FungalTraits [43] revealed contrasting ecological patterns between genome‐size groups. The increasing small‐genome taxa were primarily yeasts and two potentially pathogenic fungi (*Alternaria alternata* and *Cladosporium cladosporioides*) that became abundant in later stages. In contrast, the declining large‐genome taxa included multiple potentially pathogenic, endophytic, and saprotrophic fungi, none of which achieved dominance at any point during the study.

Unsupervised clustering of fungal community and functional attributes partitioned the temporal patterns into three groups: Group I (monotonically decreasing) includes average genome size, average transcript length, taxonomic beta diversity, and fungal OTU richness; Group II (hump‐shaped) includes community expansion rate and metatranscriptomic beta diversity; Group III (monotonically increasing) includes fungal community abundance, transcribed gene richness, maximum growth rate, average genome GC content, and average transcript GC content (Figure 1).

**FIGURE 2:**
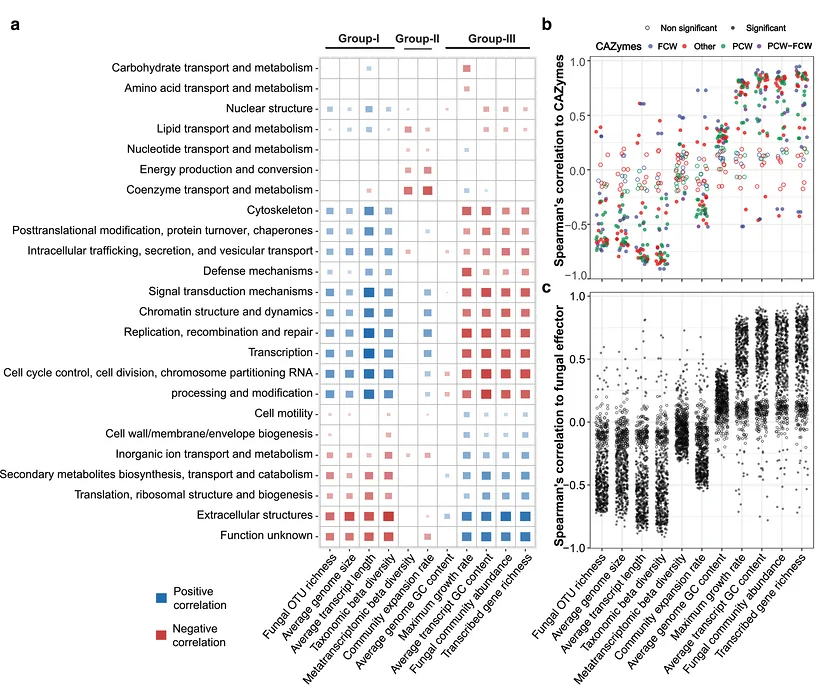
Fungal gene functions associated with fungal attributes. (a) Heatmap of associations between fungal attributes and COG (Clusters of Orthologous Groups) functional categories. Group I attributes (for example, average genome size) showed positive associations (blue) with defense mechanisms, signal transduction, replication, recombination and repair, cell cycle control, cell division and chromosome partitioning, chromatin dynamics, transcription, cytoskeleton, post‐translational modification, protein turnover and chaperones, and intracellular trafficking, secretion, and vesicular transport. Group III attributes (for example, community abundance) showed positive associations with extracellular structures, secondary metabolite biosynthesis, transport and catabolism, translation, and inorganic ion transport and metabolism. Group II attributes (particularly metatranscriptomic beta diversity) did not show a consistent positive association with any functional category. The size of each square corresponds to the absolute value of the Spearman's correlation coefficient. Blue and red colors denote positive and negative correlations, respectively. Non‐significant correlations (FDR, *p* > 0.01) are left blank (*n* = 158). (b) Spearman's correlation analyses indicated significant positive associations between Group III attributes and secreted carbohydrate‐active enzymes (CAZymes), comprising enzymes predicted to target plant cell walls (PCW) and fungal cell walls (FCW). (c) Spearman's correlation analyses indicated strong, significant positive associations between effector expression levels and Group III attributes. Open circles denote non‐significant correlations, while solid circles represent significant correlations (FDR, *p* < 0.01, *n* = 158).

The functional profile was also influenced by host development (R^2^ = 0.54, *p* < 0.001) (Figure S5). Using clusters of Kyoto Encyclopedia of Genes and Genomes (KEGG) orthology (KO) to annotate the metatranscriptomic data, we found that fungal functional orthology was also primarily categorized into three groups: monotonically decreasing, hump‐shaped, and increasing (Figure S5). Functional clusters of orthologous genes (COG) category correlations with Groups I‐III attributes revealed contrasting ecological roles. Group I attributes (monotonically decreasing) were positively associated with COG functions involved in defense mechanisms; signal transduction; replication, recombination, and repair; cell cycle control and chromosome partitioning; chromatin dynamics and transcription (Figure 2a). By contrast, Group III attributes (monotonically increasing) correlated with COG functions related to extracellular structures; secondary metabolite biosynthesis/transport/catabolism; inorganic ion transport and metabolism (Figure 2a). Group II attributes (hump‐shaped, notably metatranscriptomic beta diversity) did not show a consistent positive association with any single broad functional category (Figure 2a).

Furthermore, we analysed the temporal patterns of carbohydrate‐active enzymes (CAZymes) and secreted effectors. Among 49 secreted CAZymes, 37 (∼75.5%) associated with Group III attributes, 10 (∼20.4%) with Group II attributes, and 5 (∼10.2%) with Group I attributes (Figure 2b). The Group‐III‐associated CAZymes comprised enzymes predicted to target plant cell walls (PCW, 16), fungal cell walls (FCW, 6), combined FCW‐PCW targets (3), and other secreted activities (12) (Figure 2b). Of 572 predicted effectors, the majority (417 effectors; ∼73%) associated positively with Group III attributes, 39 associated with Group II attributes (∼6.8%), and 16 with Group I attributes (∼2.8%) (Figure 2c).

**FIGURE 3:**
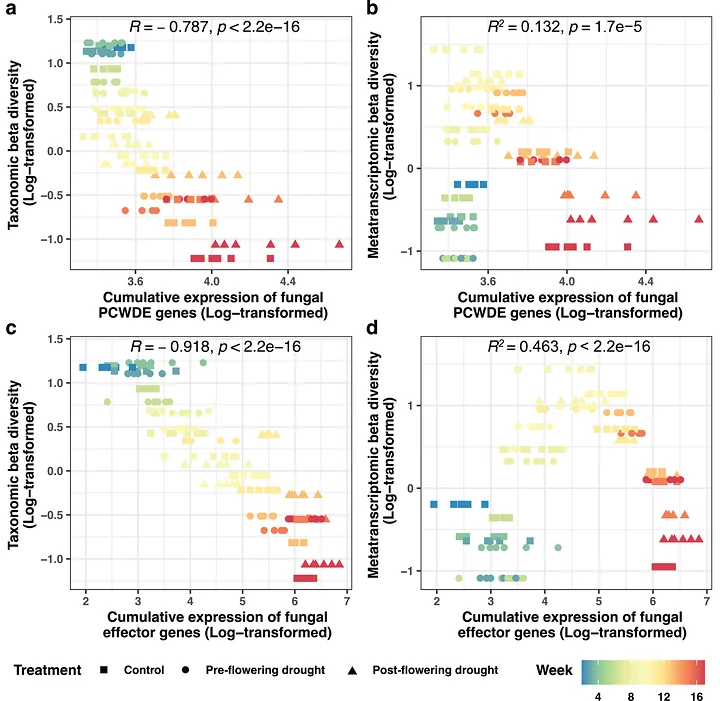
Associations between fungal beta diversity and expression of genes linked to putative pathogen activity. (a,b) Taxonomic beta diversity was strongly negatively correlated with (a) fungal plant cell wall‐degrading enzyme (PCWDE) and (b) effector genes. (c,d) Metatranscriptomic beta diversity was quadratically related to (c) fungal plant cell wall‐degrading enzyme (PCWDE) and (d) effector genes (*n* = 158).

Interestingly, we found that the expressions of fungal genes encoding secreted fungal plant cell wall‐degrading enzyme (PCWDE) and effectors are negatively correlated with fungal taxonomic beta diversity, but quadratically correlated with metatranscriptomic beta diversity (Figure 3). Residual analysis confirmed that taxonomic beta diversity alone accounts for the temporal decline in PCWDE and effector gene expression (Figure S6), suggesting that shifts in fungal community heterogeneity are particularly relevant to the expression of pathogenicity‐related genes with plant development. Given that secreted effectors and PCWDE may contribute to foliar infection by fungal pathogens [44, 45, 46], our findings indicate a link between fungal beta diversity and ecosystem function, specifically pathogen suppression.

Taken together, these results indicate that functional turnover in the leaf mycobiome is tightly linked to transient population dynamics and compositional shifts toward smaller‐genome, GC‐rich taxa that increasingly express extracellular degradation and secondary metabolic functions as the host develops. Early developmental stages are characterized instead by communities enriched in replication/repair and transcriptional machinery. These correlated patterns, rather than simple concordance between taxonomy and function, emphasize the importance of integrating both abundance‐ and expression‐based metrics to resolve mechanistic drivers of phyllosphere fungal dynamics.

### 2.2 Leaf Metabolites Linked to Fungal Functional Dynamics

We integrated untargeted leaf metabolomics from the same time‐series samples with the fungal community and metatranscriptomic datasets to identify metabolites whose temporal abundance covaried with fungal functional attributes. We calculated leaf metabolic beta diversity and tested its correlation with fungal taxonomic and functional beta diversity. Leaf metabolic beta diversity showed no significant temporal dynamics and was uncorrelated with either fungal taxonomic or functional beta diversity (Figure S7).

**FIGURE 4:**
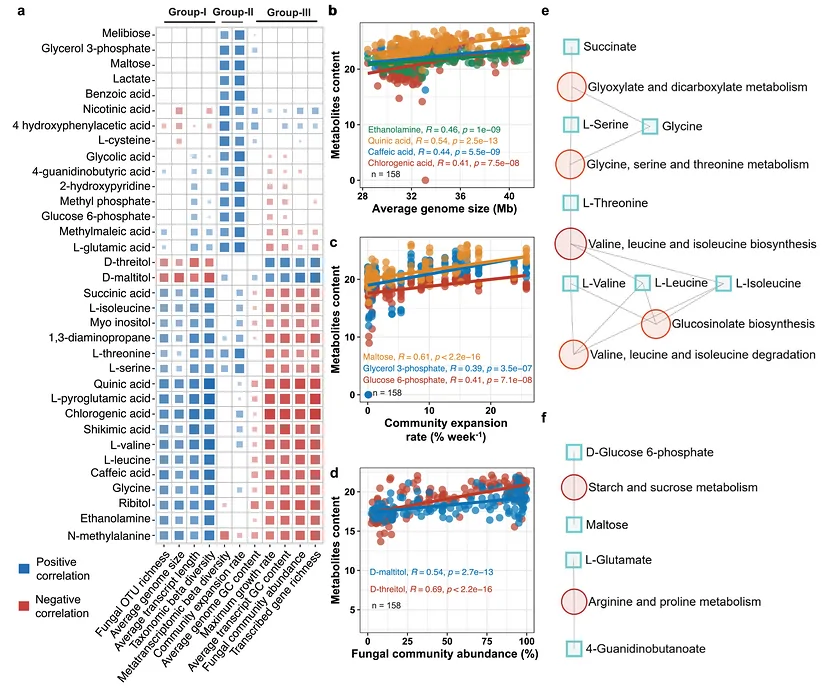
Correlations between fungal attributes and sorghum metabolites. (a) Heatmap of associations between fungal attributes and sorghum metabolites. Group I attributes showed positive correlations (blue) with 17 metabolites, including phenolic acids such as chlorogenic acid, caffeic acid, and quinic acid, and branched‐chain amino acids (leucine, isoleucine, valine). Group II attributes showed positive correlations with 15 metabolites, primarily labile carbohydrates and phosphorylated sugar derivatives (maltose, glycerol‐3‐phosphate, glucose‐6‐phosphate). Group III attributes were positively correlated with 2 metabolites, both sugar alcohols (maltitol, threitol). The size of each square corresponds to the absolute value of Spearman's correlation coefficient. Blue and red colors denote positive and negative correlations, respectively. Non‐significant correlations (FDR, *p* > 0.01) are left blank (*n* = 158). (b) The average genome size of Group I attributes was positively correlated with concentrations of broad‐spectrum antifungal chemicals, such as chlorogenic acid, caffeic acid, quinic acid, and ethanolamine. (c) Community expansion rate of Group II attributes was positively correlated with concentrations of resource metabolites, such as maltose, glycerol‐3‐phosphate, and glucose‐6‐phosphate. (d) Fungal community abundance of Group III attributes was positively correlated with concentrations of maltitol and threitol. Correlations between sorghum metabolites and fungal attributes were assessed using Pearson's correlation coefficient. (*n* = 158). (e) An enrichment network of interconnected metabolic pathways based on the 17 metabolites positively correlated with Group I attributes. (f) An enrichment network of interconnected metabolic pathways based on the 15 metabolites positively correlated with Group II attributes. Circular nodes represented metabolic pathways identified as significant by a hypergeometric test (*p* < 0.01). Node color corresponded to enrichment *p*‐value, with darker red indicating higher significance. Node size was proportional to the number of metabolites from our dataset found in that pathway. Square nodes represented related metabolites.

Using Spearman's rank correlations (*ρ* > 0.6) with false‐discovery‐rate correction (FDR, *p* < 0.01), we detected 34 metabolites that were robustly associated with the three aforementioned temporal attribute groups (Group I: monotonically decreasing; Group II: hump‐shaped; Group III: monotonically increasing) (Figure 4). Seventeen metabolites exhibited a positive correlation with Group I traits (e.g., decreasing genome size) but a negative correlation with Group III traits (e.g., increasing community abundance) (Figure 4). These seventeen metabolites include a number of phenolic acids (chlorogenic acid, caffeic acid, and quinic acid) and branched‐chain amino acids (leucine, isoleucine, and valine) (Figure 4). Metabolite pathway enrichment analysis of these seventeen metabolites revealed a significant enrichment of functions associated with the synthesis and metabolism of branched‐chain amino acids (Figure 4e). Notably, these seventeen metabolites included known broad‐spectrum antifungal compounds such as chlorogenic acid [47], caffeic acid [48], quinic acid [49], ethanolamine [50], and branched‐chain amino acids (via inducing apoptosis‐like cell death) [51]. This inverse relationship suggests that antifungal metabolites simultaneously suppress overall fungal community expansion (Group III) and favor taxa characterized by larger genomes (Group I), potentially reflecting enhanced tolerance mechanisms.

Although only two metabolites showed a positive association with Group III traits and a negative association with Group I traits, both are sugar alcohols‐maltitol and threitol. Prior work has demonstrated that adding sugar alcohols can significantly alter soil microbial communities and enzyme activities [52, 53, 54]. Specifically, sugar alcohols such as sorbitol and mannitol significantly increase soil bacterial community abundance and diversity, and enhance activities of multiple enzymes involved in carbon, nitrogen, and phosphorus cycling, including urease, catalase, alkaline phosphatase, β‐glucosidase, and N‐acetyl‐β‐d‐glucosaminidase; the magnitude and type of these effects are concentration‐ and compound‐dependent [52, 53, 54]. Additionally, threitol has been found to specifically function as an important metabolic signal during the eucalypt‐*Armillaria* interaction before infection [55].

Fifteen metabolites correlated specifically with Group II attributes, notably the hump‐shaped community expansion rate (Figure 4). These metabolites were dominated by labile carbohydrate and phosphorylated sugar derivatives (for example, maltose, glycerol 3‐phosphate, and glucose 6‐phosphate) that represent readily utilizable carbon and phosphate sources for microbes (Figure 4). Metabolite pathway enrichment analysis of these fifteen metabolites revealed a significant enrichment of functions associated with starch and sucrose metabolism (Figure 4f). The temporal peak of these substrates around flowering aligns with the transient proliferation of fast‐growing fungal taxa, consistent with a resource‐pulse driven bloom of taxa with high growth rates and expression of biosynthetic/energy metabolism pathways.

In our study, we attribute the detected leaf metabolites primarily to plant origin. Prior studies have established that leaf metabolites are predominantly plant‐derived [56]. Our transcriptomic data support this assumption: fungal transcripts constituted less than 1% of total transcripts, indicating minimal fungal biomass [9]. Furthermore, our untargeted metabolomic approach detected only abundant, commonly occurring metabolites. Given the relatively low fungal biomass, these high‐concentration metabolites are most parsimoniously explained by plant rather than fungal biosynthesis. While we cannot definitively exclude all fungal contributions, the evidence strongly indicates that the detected metabolome is predominantly plant‐derived.

### 2.3 Holo‐omic Integration

**FIGURE 5:**
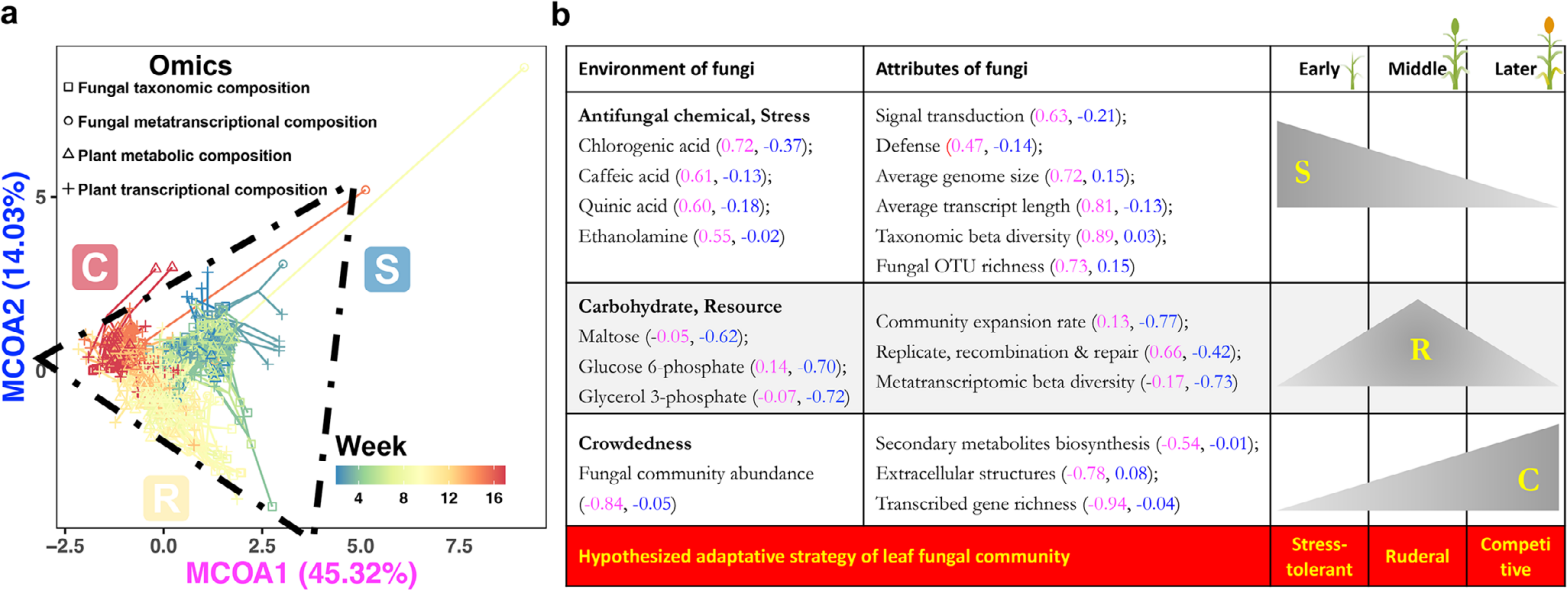
Synthesis. (a) Dimensionality reduction. Multiple co‐inertia analysis (MCOA) integrated holo‐omics datasets, including fungal community composition, fungal metatranscriptomics, plant transcriptomics, and plant metabolomics. The first two MCOA axes account for 59.35% of the total variance. The biplot revealed a temporal gradient aligned with plant developmental stages: early stage (MCOA1 > 0, MCOA2 > 0), flowering stage (MCOA1 ≈ 0, MCOA2 < 0), and late stage (MCOA1 < 0, MCOA2 > 0) (*n* = 158). (b) Temporal adaptive trajectory. Early stage fungi exhibited a stress‐tolerant (S) strategy, likely filtered by plant antifungal metabolites. They have larger genomes, longer transcripts, and functions enriched for signal transduction and defense. Flowering stage fungi displayed a ruderal (R) strategy, presumably promoted by transient nutrient availability (for example, maltose), with traits associated with rapid growth. Late stage fungi exhibited a competitive (C) strategy, with enhanced functions including secondary metabolite biosynthesis, effector deployment, secreted carbohydrate‐active enzymes (CAZymes), and extracellular structures, traits beneficial in crowded, competitive communities. For each attribute, the first number (pink) denoted the Spearman correlation with MCOA1 and the second number (blue) denoted the Spearman correlation with MCOA2.

To capture coordinated dynamics across the plant‐fungal system, we conducted multiple co‐inertia analysis (MCOA) on four data matrices (fungal taxonomic composition, fungal metatranscriptional composition, plant transcriptional composition, and plant metabolic composition) (Figure 5; Figure S8). The first two MCOA axes explained 45.32% (MCOA1) and 14.03% (MCOA2) of total co‐variation (Figure 5; Figure S9). MCOA1 correlated with antifungal compounds (e.g., chlorogenic acid) positively and fungal community abundance negatively, while MCOA2 correlated negatively with nutrient substrates (e.g., maltose) and fungal community expansion rate (Figure 5). Crucially, the MCOA biplot revealed a clear temporal gradient corresponding to plant development stages: early stage (MCOA1 > 0; MCOA2 > 0), flowering stage (MCOA1 ≈ 0; MCOA2 < 0), and late stage (MCOA1 < 0; MCOA2 > 0) (Figure 5), demonstrating that metabolite shifts of developmental host are tightly coupled to shifts in fungal functional states.

Taken together, the metabolite‐functional associations support a three‐phase adaptive sequence in the leaf mycobiome across host ontogeny. First, early leaves are enriched in antifungal/defence metabolites and select for stress‐tolerant taxa with larger genomes and elevated genome‐maintenance and signaling functions (S‐like strategy) (Figure 5). Second, a subsequent pulse of labile carbon/phosphorylated substrates around flowering promotes transient proliferation of fast‐growing ruderal taxa (R‐like strategy), producing the observed hump in community expansion rate and metatranscriptomic turnover (Figure 5). Third, in later development, as community density increases and accessible substrates per capita decline, competitor‐like taxa (C‐like) characterized by smaller genomes, enriched secondary metabolism, secreted CAZymes, and effectors become dominant. These correlated patterns are consistent with a non‐canonical S‐R‐C adaptive trajectory in which host chemical environment and resource pulses act as primary drivers of fungal functional succession.

## 3 Discussion

Our results indicate that temporal changes in the leaf metabolome are a primary driver of sequential shifts in phyllosphere fungal community composition and functional attributes, yielding a noncanonical successional trajectory from stress‐tolerators (S) via ruderals (R) to competitors (C) (Figure 5). Below, we synthesize the mechanistic links suggested by our data and discuss ecological implications and future directions.

### 3.1 Early Leaves — Chemical Filtering and Enrichment of Stress‐Tolerant Fungi

Young leaves are chemically defended and act as strong environmental filters. In particular, higher concentrations of antifungal compounds in early leaves were associated with (i) the survival of fungi with larger genomes and (ii) elevated transcription of signal transduction and regulatory genes (Figures 1−4). These patterns are consistent with a scenario in which strong biotic selection by host metabolites favors fungal taxa that carry expanded sensory and regulatory repertoires—genomic features that facilitate detection of, and transcriptional responses to, chemical stressors. Analogous patterns have been reported for bacteria under abiotic stress (acidic soil pH, salinity, heavy metals, and concurrent multiple stresses) [16, 17, 57, 58]; our data suggest that the same principle applies to fungal assemblages and that host‐derived (biotic) stressors can similarly act as abiotic stress in selecting for larger, more regulatory‐rich genomes.

### 3.2 Mid Season (flowering) — Resource Pulses Favor Ruderal Strategies

During flowering, we observed elevated levels of labile carbon (for example, maltose) together with reduced concentrations of antifungal compounds, and a concomitant increase in fungal community expansion rate (Figures 1−4). This combination of increased resource availability and weakened chemical selection is consistent with enrichment for fast‐growing, ruderal (R‐strategy) fungi that invest in rapid biomass production rather than extensive stress‐response machinery. The pattern also mirrors soil systems where resource‐rich conditions increase potential microbial growth rates (e.g., ^18^O incorporation studies [59]). Thus, transient pulses of readily metabolizable substrates in the leaf environment appear to shift trait distributions toward growth‐oriented strategies.

### 3.3 Late Stage — Density‐Dependent Antagonism and Competitive Traits

As fungal abundance increases later in the season, we detected positive correlations between fungal community abundance and expression of genes implicated in antagonism‐secondary metabolite biosynthesis, secreted CAZymes that can target fungal cell walls, and candidate effector proteins (Figures 1−4). These signatures indicate an intensification of biotic interactions and interference competition as communities become crowded, with taxa allocating resources to antagonistic and defensive functions.

Note the effectors and secreted CAZymes might also target plant cells. As previously reported, both leaf microbiota and age drive a mechanism that balances the growth‐defense trade‐off in individual leaves [60]. The temporal increase in expression of these effector and secreted CAZymes suggest that once a plant has reproduced, it may therefore experience a reduced selective advantage in allocating resources toward fungal resistance mechanisms, such as the synthesis of antifungal compounds or the enhancement of systemic immune responses.

Metabolites mediate bidirectional plant‐fungal interactions: plants deploy metabolites to filter and modulate fungal colonists, while fungi reciprocally influence plant physiology through their secondary metabolites. Our study focuses on the plant‐to‐fungus direction, revealing how the leaf metabolome shapes fungal functional dynamics. Our approach does not distinguish plant‐derived from fungal‐derived metabolites, though prior studies suggest fungal metabolic signatures are barely detectable in leaves [56]. The reciprocal pathway about how fungal secondary metabolites modulate plant phenotypes remains a key knowledge gap that will be important for harnessing the microbiome toward resilient and sustainable crops.

### 3.4 Decoupling of Taxonomic and Functional Beta Diversity

An interesting outcome of our analyses is the temporal dissociation between taxonomic and functional beta diversity. Functional beta diversity peaked during flowering, coincident with reduced antifungal selection and increased resource abundance, whereas taxonomic beta diversity was highest in early small communities (consistent with ecological drift [9]). This pattern implies that different assembly processes act on the two dimensions: reduced selection by resource limitation and antifungal metabolite increases functional divergence among communities, while small community abundance and stochastic processes inflate taxonomic overdispersion. Prior work has identified release from selection and small community abundance as drivers of increased beta dispersion [4, 61, 62, 63, 64]; our findings extend these ideas by showing that these mechanisms can operate simultaneously but differentially affect taxonomic vs. functional beta diversity.

Interestingly, as plants matured and both fungal taxonomic and functional beta diversities declined, we observed an upregulation of fungal genes associated with pathogenicity, including effectors and secreted CAZymes targeting the plant cell wall as well as the fungal cell wall (Figure 3), indicating a link between fungal beta diversity and pathogen activity suppression. Along with this process, *Cladosporium cladosporioides* [65] and *Alternaria alternata*, two taxa that include many documented plant pathogens, increased in relative abundance and dominated the leaf fungal communities as plants matured. This finding indicates a link between fungal beta diversity and potential pathogen activity suppression.

Notably, our detection of a decoupling between taxonomic and functional beta diversity in fungi extends recent findings of decoupling in taxonomic and functional alpha diversities of bacterial communities at local and regional scales [17, 66]. This recurring disconnect across kingdoms raises critical questions about how different components (taxonomic vs. functional; alpha vs. beta) of diversity contributes to ecosystem function and underscores the necessity of trait‐based approaches in microbial ecology [13].

Beyond the pronounced temporal dynamics, the drought treatment exerted a modest impact on fungal functional attributes. The drought effect primarily operated through its influence on fungal relative abundance, which in turn affected the derived community expansion rate and, consequently, fungal functional beta diversity (Figure 1). Specifically, pre‐flowering drought delayed the development of fungal abundance, community expansion rate, and fungal functional beta diversity by approximately two weeks. Importantly, this delay was not offset by rewetting after the drought period, indicating a persistent legacy effect of early‐season water limitation on fungal community dynamics. These findings add to the growing body of evidence highlighting the impact of global change drivers on phyllosphere microbiomes and underscore the importance of considering legacy effects when predicting microbial responses to climate variability [67].

## 4 Conclusions

This study suggests a novel adaptive trajectory (S‐R‐C) for host‐associated fungal communities by directly linking plant metabolomes with fungal functional traits. Our findings challenge the conventional r‐ to K‐ succession model based on inference of bacterial growth rate [19, 68], and provide a mechanistic understanding of fungal community assembly over time. The holo‐omics approach was pivotal to this discovery, enabling us to identify key plant metabolites and fungal traits involving the succession of S‐, R‐, and C‐strategists: early‐leaf antifungal metabolites filter S‐strategists with large genomes; a transient pulse of maltose at flowering triggers the proliferation of fast‐growing R‐strategists; and in mature leaves, C‐strategists utilize secondary metabolism to thrive in crowded, competitive environments.

Our study has implications for crop pathogen management. We found that pathogen‐related gene expression negatively correlated with fungal beta diversity, indicating the importance of maintaining fungal community and functional heterogeneity. This can be achieved through multiple strategies: crop breeding to enhance production of antifungal compounds, or exogenous addition of these metabolites, and agricultural management practices such as staggered planting, intercropping, and diversified field microhabitats. Additionally, to prevent late‐stage competitive dominance by small‐genome, potential pathogenic taxa such as *Alternaria*, we may consider introducing beneficial small‐genome competitors or biocontrol agents.

## 5 Experimental Section

### 5.1 Datasets and Sequence Processing

This study integrates and analyzes leaf multi‐omics datasets from a semiarid agricultural field that were previously published across several studies [9, 39, 69]. Briefly, leaf, root, rhizosphere, and soil samples were collected weekly throughout a growing season from sorghum plants subjected to three irrigation regimes: a continuously irrigated control, pre‐flowering drought, and post‐flowering drought [9, 39, 69]. The present work focuses on integrating leaf‐derived data, including RNA‐seq, metabolomics, and ITS2 metabarcoding, to address how the leaf metabolome influences fungal functional dynamics.

The raw RNA‐seq reads were first quality‐controlled using fastp v0.12.4 [70]. High‐quality reads were then aligned to the *Sorghum bicolor* v3.1.1 transcript reference (Phytozome v13, https://phytozome‐next.jgi.doe.gov/info/Sbicolor_v3_1_1) using bowtie2 v2.5.3 [71] to identify and remove sorghum sequences. The remaining non‐sorghum reads were further filtered to eliminate rRNA sequences using ribodetector v0.2.7 [72]. Subsequently, the filtered reads were assembled into contigs with MEGAHIT v1.2.9 [73], employing a range of k‐mer sizes (29, 39, 59, 79, 99, 119, and 141). Contigs shorter than 500 bp were discarded. The retained contigs were then classified as eukaryotic using EukRep v0.6.7 [74], and redundant sequences within these eukaryotic contigs were reduced with CD‐HIT v4.8.1 [75]. These curated eukaryotic contigs were annotated by alignment to a fungal non‐redundant database (version 4751). Expression levels of fungal genes were quantified using Salmon v1.10.1 [76], with abundances reported in transcripts per million (TPM) to normalize for gene length and sequencing depth differences. Functions of these fungal genes were annotated by referencing the EggNOG v5.0.2 database [77] using DIAMOND v2.1.8 [78]. From the fungal genes, secreted proteins were predicted using SignalP v6.0 (threshold: Sec/SPI > 0.8) [79]. Subsequently, EffectorP v3.0 [80] was employed to identify putative effectors from these secreted proteins, and carbohydrate‐active enzymes (CAZymes) were detected by alignment against the CAZy database.

### 5.2 Calculation of Fungal Functional Attributes

We then computed a suite of fungal functional attributes from the fungal metatranscriptomic dataset. Transcribed fungal gene richness was defined as the number of fungal genes detected with expression in each sample. The average transcript length was calculated by weighting the length of each transcript by its relative abundance. Similarly, the average transcript GC content was computed using the GC content of each transcript, weighted by its abundance. Metatranscriptomic beta diversity was defined as the homogeneity in fungal gene expression profiles across samples, beta diversity was assessed based on Bray‐Curtis distances of the fungal metatranscriptional composition using the betadisper function in the vegan package v2.6‐4 in R [81]. Additionally, the maximum growth rate was estimated from codon usage bias using gRodon v2.5.1 [82].

The community expansion rate (g) was derived from the derivative of a logistic growth curve fitted to the temporal dynamics of fungal community abundance (g = dN/dt). OTUs from ITS2 metabarcoding were assigned to functional guilds by matching their taxonomic identities against the FungalTraits database [43]. Fungal genome size and GC content were calculated by integrating OTU taxonomic identities with genomic trait data retrieved from NCBI (Table S1). For each sample, trait values (genome size or GC content) of individual OTUs were weighted by their relative abundances and summed to yield the community‐level average. To infer the temporal dynamics of each fungal attribute and fungal OTU, we fitted a regression model for each fungal attribute and fungal OTU against sample time.

### 5.3 Statistical Analysis

We conducted Spearman's rank correlation analyses using the psych package v2.3.3 [83] to assess associations among fungal attribute groups, fungal clusters of orthologous genes (COG) categories, and sorghum metabolites. Multiple testing was controlled using the Benjamini–Hochberg procedure, with significance defined as false discovery rate (FDR) *p* < 0.01. Pairwise relationships were visualized as a correlation heatmap in ggplot2 v3.4.1 [84]. Based on correlations between fungal attribute groups and sorghum metabolites, we identified metabolites significantly associated with specific attribute groups. To contextualize metabolite function, we performed enrichment network of interconnected metabolic pathways using a hypergeometric test against the *Zea mays* KEGG pathway library in MetaboAnalyst v6.0 [85].

Following Hellinger transformation of all datasets, principal coordinates analysis (PCoA), followed by permutational multivariate analysis of variance (PERMANOVA), was used to test and visualize variation in fungal metatranscriptional composition based on Bray–Curtis dissimilarities, using the ape v5.7‐1 [86] and vegan v2.6‐4 packages [81]. To assess pairwise associations between fungal metatranscriptional composition and each of the other compositional datasets (fungal taxonomic composition, plant transcriptional composition, and plant metabolic composition), we performed Procrustes analyses on the PCoA ordinations and Mantel tests on Bray–Curtis dissimilarity matrices with ecodist v2.0.9 [87] and vegan v2.6‐4 [81]. Multiple co‐inertia analysis (MCOA) was conducted with ade4 v1.7‐22 [88] to explore multivariate relationships among these datasets. Threshold Indicator Taxa Analysis (TITAN) was applied with TITAN v2.4.3 [89] to identify taxa showing significant temporal change and the corresponding change points. All analyses were performed in R v4.2.3, except metabolite pathway enrichment, which was conducted with MetaboAnalyst [85].

## Author Contributions

P.C., C.G., and J.W.T. conceived of and wrote the manuscript. P.C. and C.G. performed the bioinformatics and statistical analyses.

## Code Availability

The source code implementing the analyses in this manuscript will be available on Github https://github.com/chenpl2023/Leaf_multi‐omic.

## Conflicts of Interest

The authors declare no conflict of interest.

## Supporting information




**Supporting File**: advs74875‐sup‐0001‐SuppMat.pdf.

## Data Availability

No new data were generated in this study. All seqence data have been previously deposited in NCBI (i) Sequence Read Archive (fungal ITS2 metabarcoding) with accession codes Bioproject PRJNA412410 and PRJNA494573, (ii) Gene Expression Omnibus database (Sorghum transcriptome and fungal metatranscriptome) with accession code GSE12844. Metatabolome data have been previously deposited in MassIVE with accession code MSV000081639.
