# Supplementary material for: Linking Plant Metabolomics with Fungal Functional Dynamics Reveals a Noncanonical S‐R‐C Adaptive Trajectory
Source: Adv Sci (Weinh). 2026 Mar 18;13(31):e74875. doi: 10.1002/advs.74875 (PMC13587999; doi:10.1002/advs.74875)
Supplement: Supplementary file 1 [file ADVS-13-e74875-s001.pdf]

Supplementary Materials for

Linking plant metabolomics with fungal functional dynamics reveals a noncanonical S-R-C adaptive trajectory

Peilin Chen, *John W. Taylor*, *Cheng Gao*

This PDF file includes:

Figures S1-S9

Tables S1

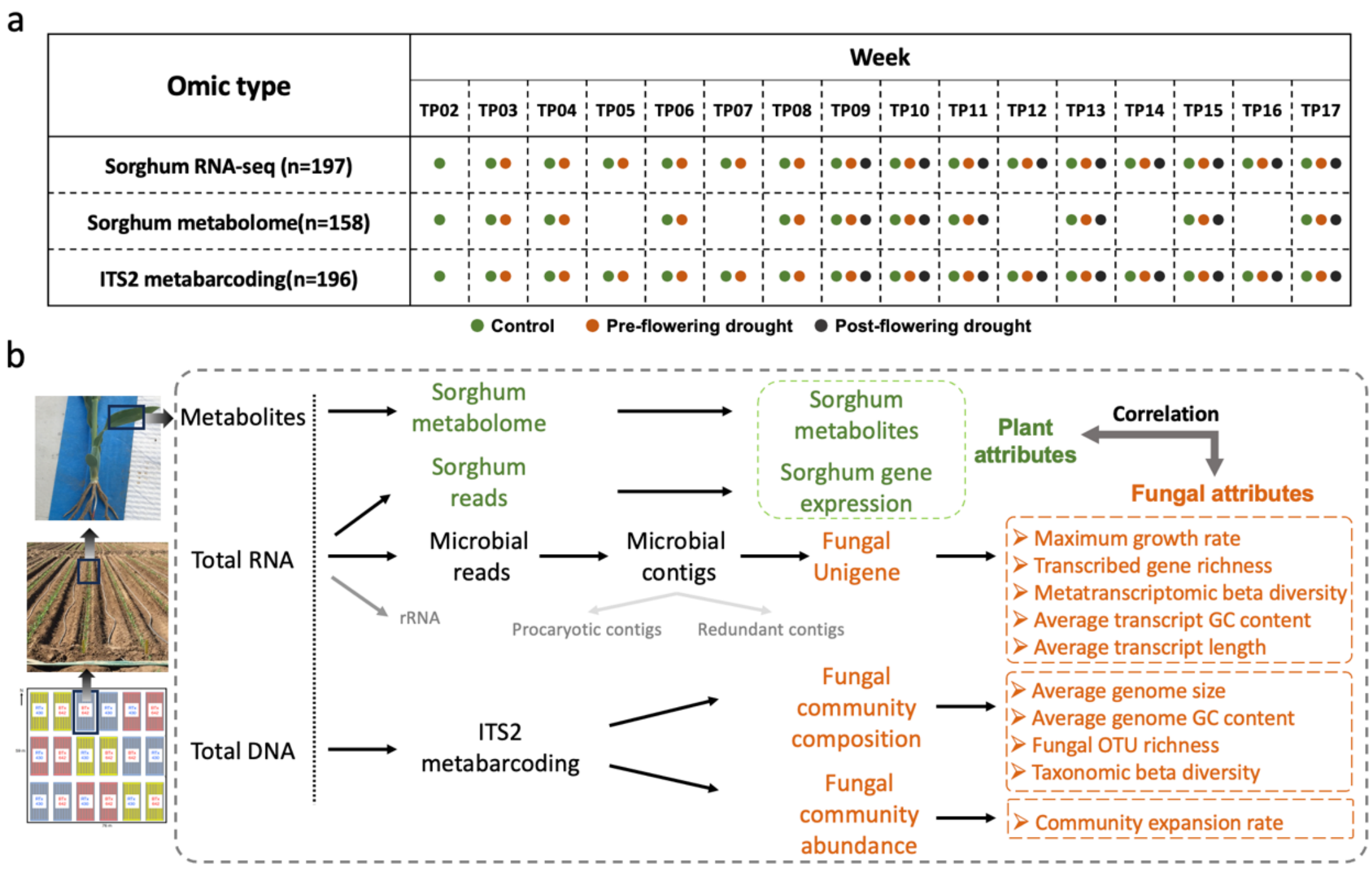

Figure S1. Multi-omics datasets and analysis workflow. a We integrated leaf mRNA-seq, fungal ITS2 metabarcoding, and untargeted leaf metabolomics derived from previously published field datasets [1-3]. **b** Leaf mRNA-seq comprised both the plant transcriptome and the fungal metatranscriptome because plant and fungal mRNAs share poly(A) tails. We therefore extracted fungal reads from the leaf mRNA-seq data to assess fungal gene expression and the average transcript length of the fungal community. The average fungal genome size was estimated by matching ITS2-assigned taxa to a fungal genome database compiled from NCBI.

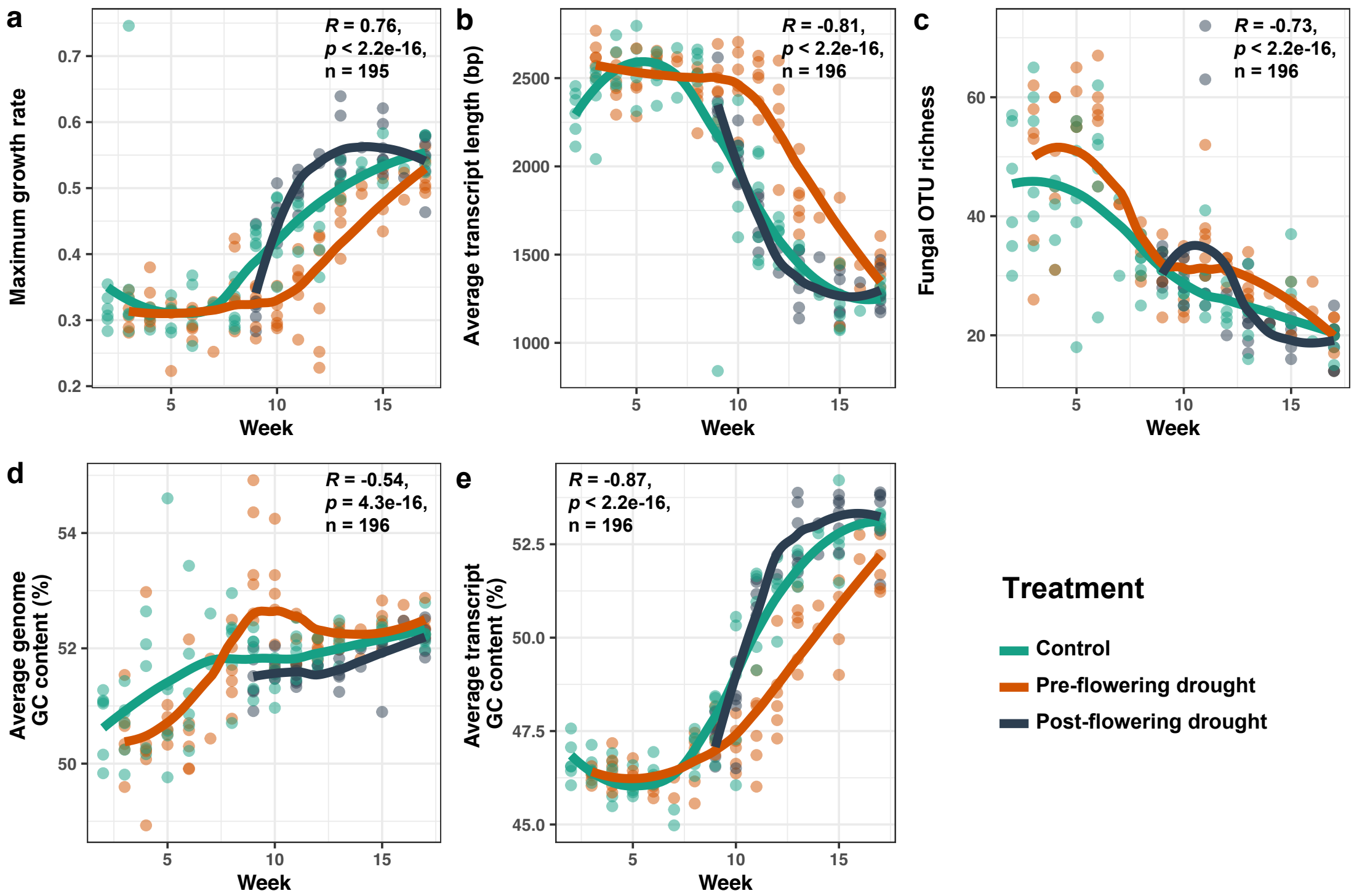

Figure S2. Temporal dynamics of fungal attributes: a maximum growth rate, **b** average transcript length, **c** operational taxonomic unit (OTU) richness, **d** average genome GC content, and **e** average transcript GC content. This figure supplemented the temporal dynamics of fungal attributes shown in Figure 1.

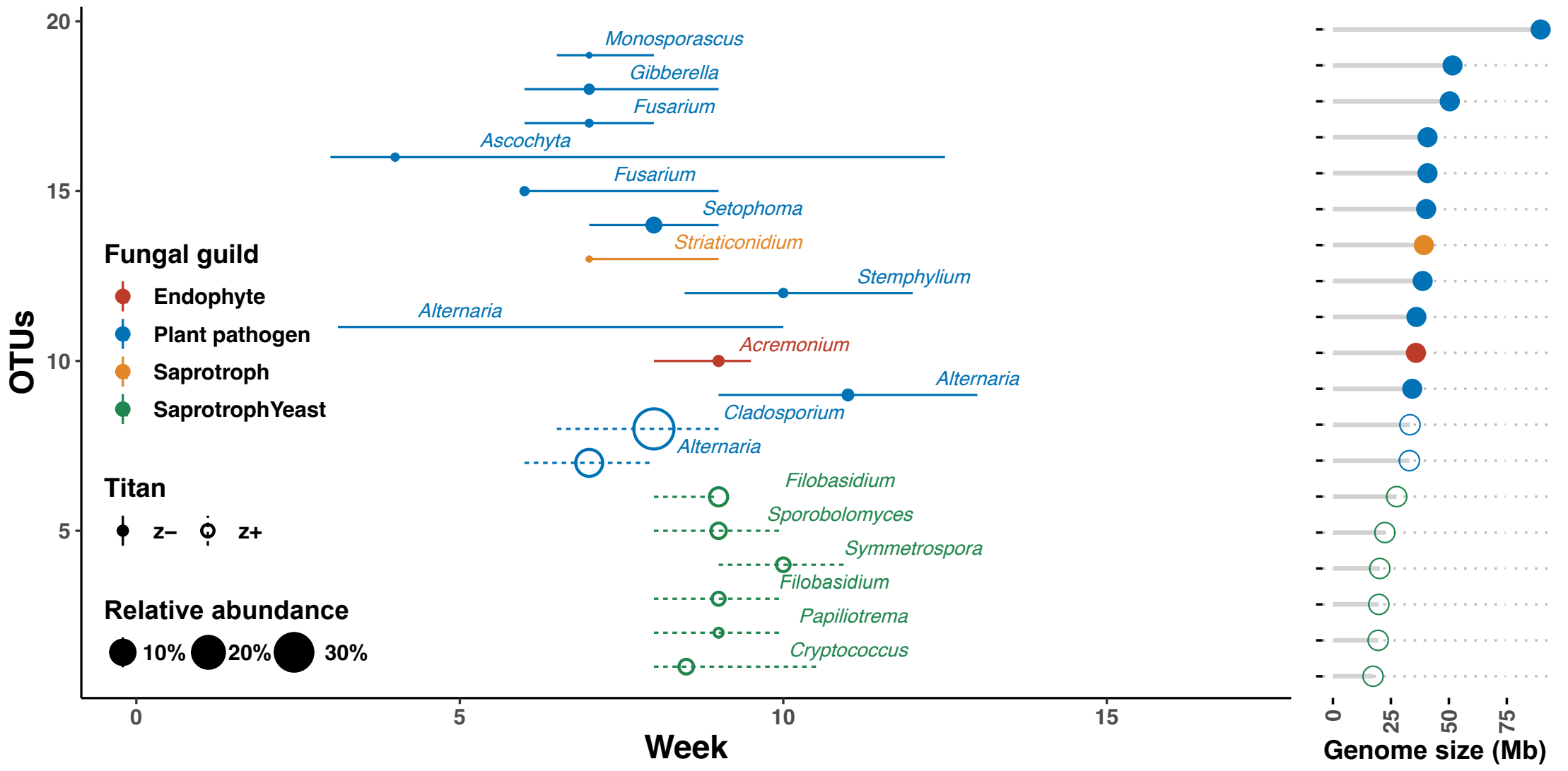

Figure S3. Threshold indicator taxa analysis of the top 25 most abundant fungal operational taxonomical units (OTUs). Threshold indicator taxa analysis (TITAN) showed that the decrease in fungal average genome size was driven by an increase in the relative abundance of small-genome fungal taxa (17.3-33.2 Mb), led by six Basidiomycota yeasts and two Ascomycota filamentous fungi, and by a significant drop in the relative abundance of large-genome taxa (34.2-89.6 Mb), led by ten Ascomycota taxa. For each taxon, functional guilds were differentiated by color; open symbols indicated increasing relative abundance (z+), and filled symbols indicated declining relative abundance (z−) (n = 196).

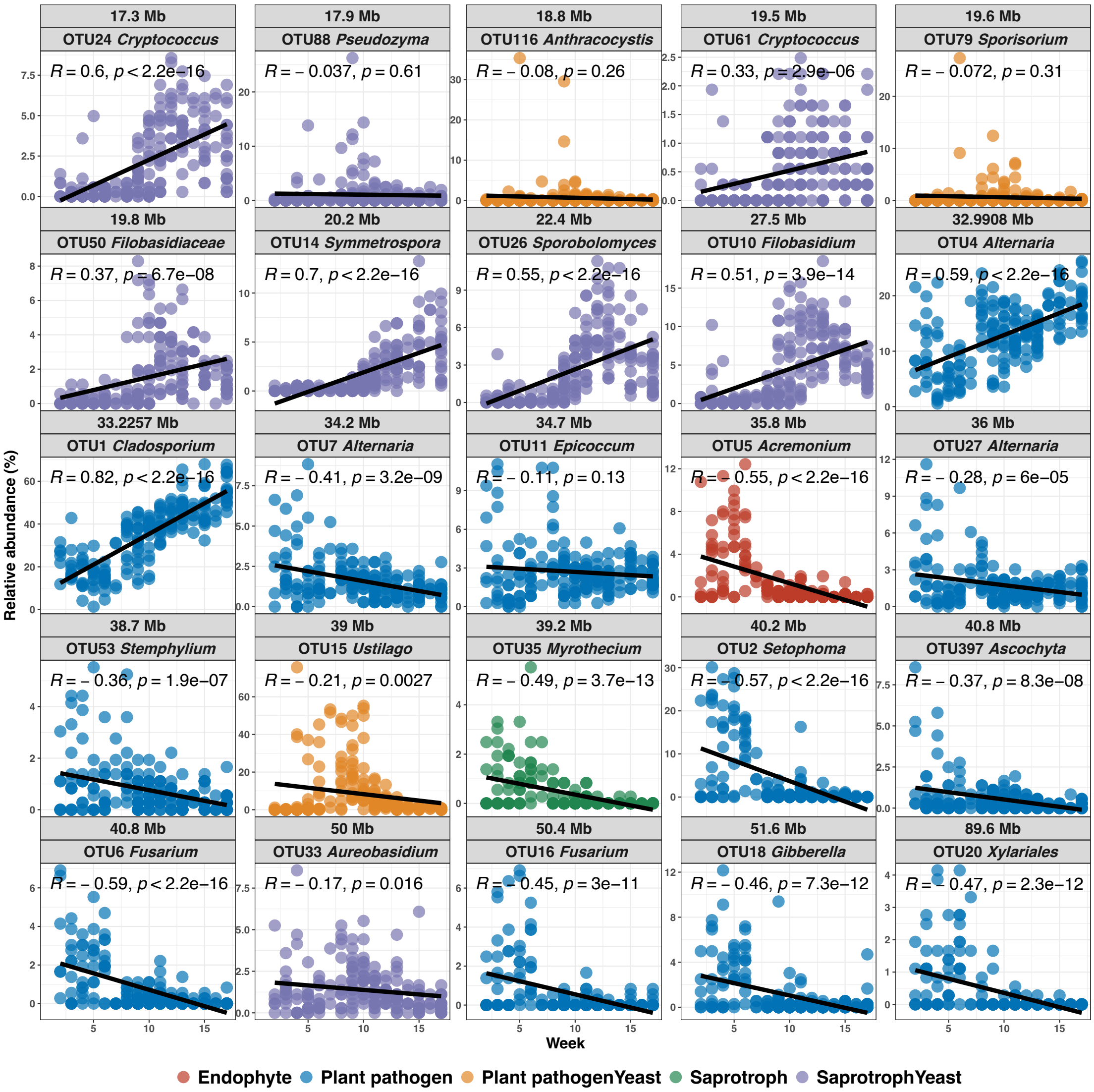

Figure S4. Temporal dynamics of the top 25 most abundant fungal operational taxonomical units (OTUs). Different colors represented different fungal guilds Correlations were assessed using Pearson's correlation coefficient (n = 196).

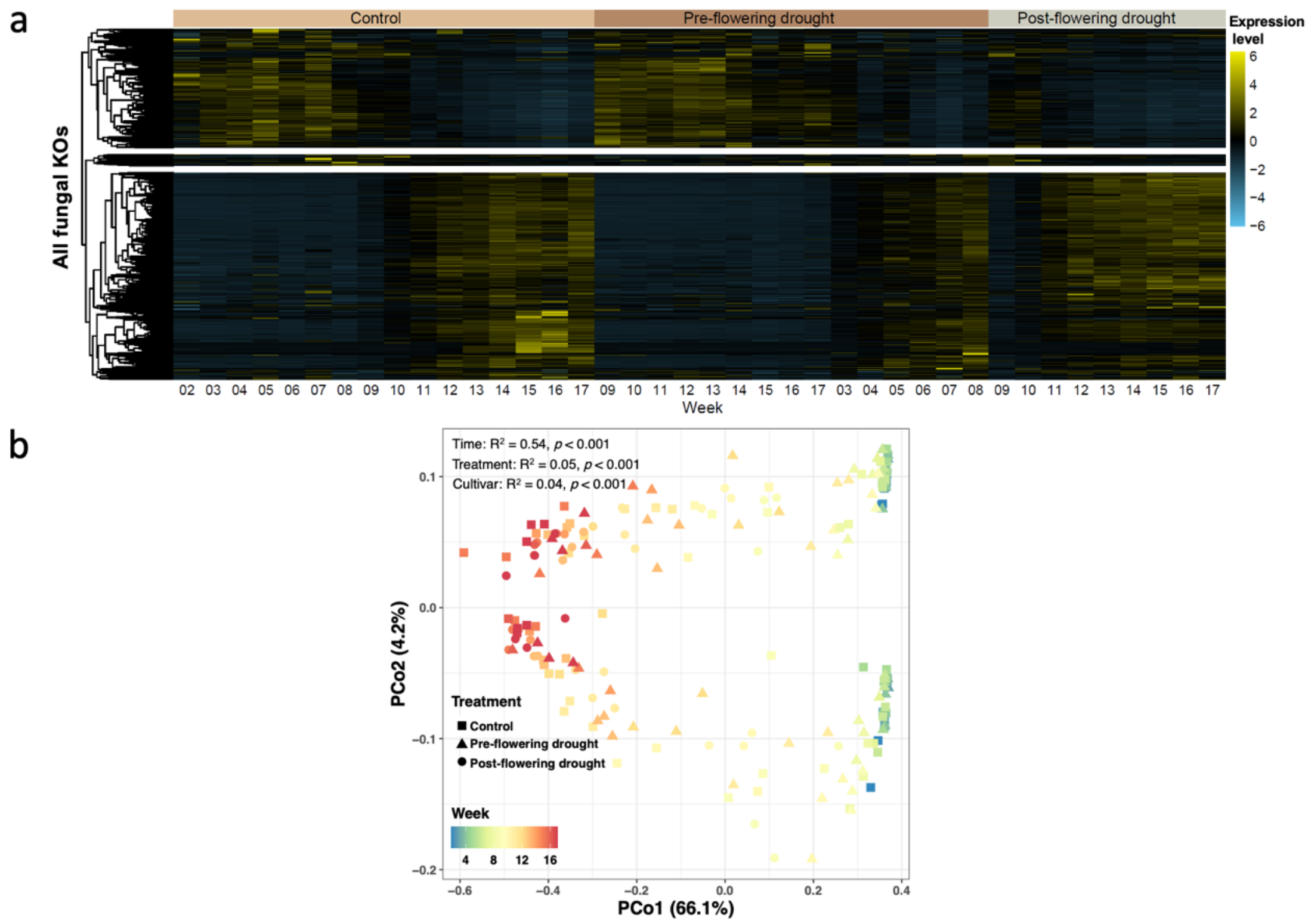

Figure S5. Temporal dynamics of fungal metatranscriptomic composition. a The heatmap showed the temporal changes in relative abundances of Kyoto Encyclopedia of Genes and Genomes (KEGG) Orthologs (KOs) in fungal metatranscriptome along with plant development. **b** Fungal KO composition in leaf was influenced by, in order of importance, time, treatment and host cultivar, based on principal coordinate (PCo) analysis with permutational analysis of variance (PERMANOVA) (n = 196).

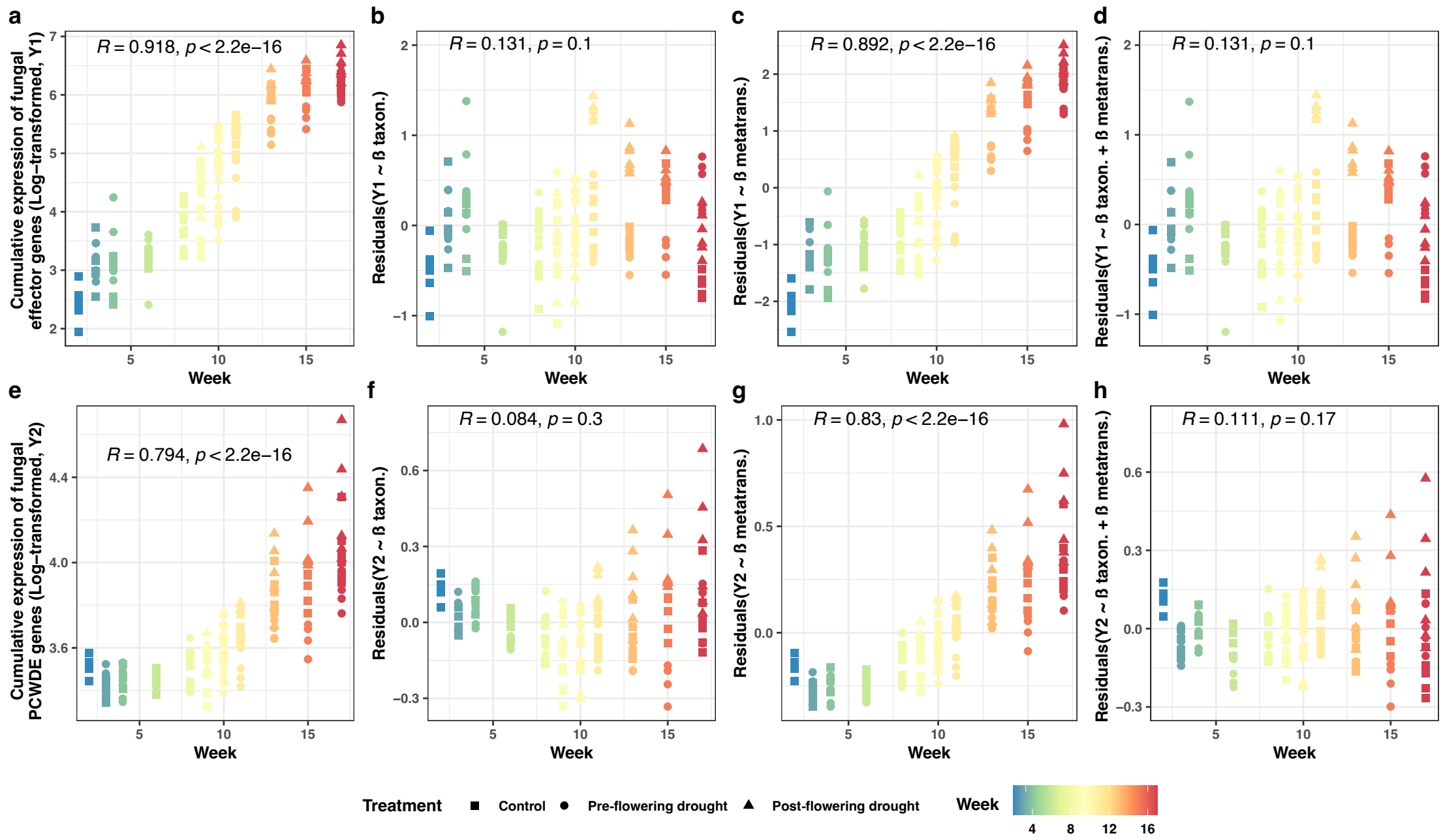

Figure S6. Temporal distribution of putative pathogen effector genes and their residuals after modeling with fungal beta diversity. a–d, Fungal effectors. **a**, Temporal distribution of expression levels for genes encoding fungal effectors (Y1). **b**, Residuals from effector expression modeled against fungal taxonomic beta diversity. **c**, Residuals from effector expression modeled against fungal metatranscriptomic beta diversity. **d**, Residuals from effector expression modeled against both fungal taxonomic and metatranscriptomic beta diversity. **e–h**, plant cell wall-degrading enzyme (PCWDE) (Y2). **e**, Temporal distribution of expression levels for genes encoding PCWDE (Y2). **f**, Residuals from PCWDE expression modeled against fungal taxonomic beta diversity. **g**, Residuals from PCWDE expression modeled against fungal metatranscriptomic beta diversity. **d**, Residuals from PCWDE expression modeled against both fungal taxonomic and metatranscriptomic beta diversity.

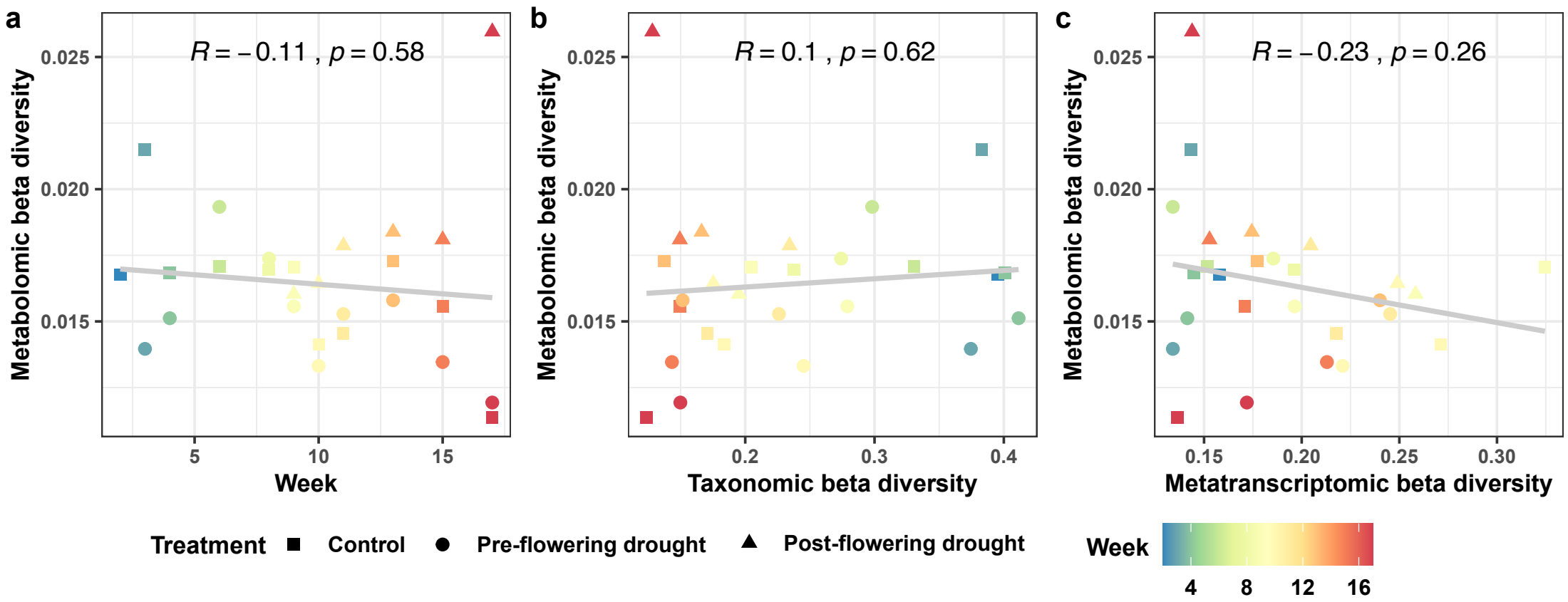

Figure S7. Leaf metabolomic beta diversity showed **a** no significant temporal dynamics and no significant correlation with fungal **b** taxonomic or **c** functional beta diversity. Correlations were assessed using Pearson's correlation coefficient (n = 27).

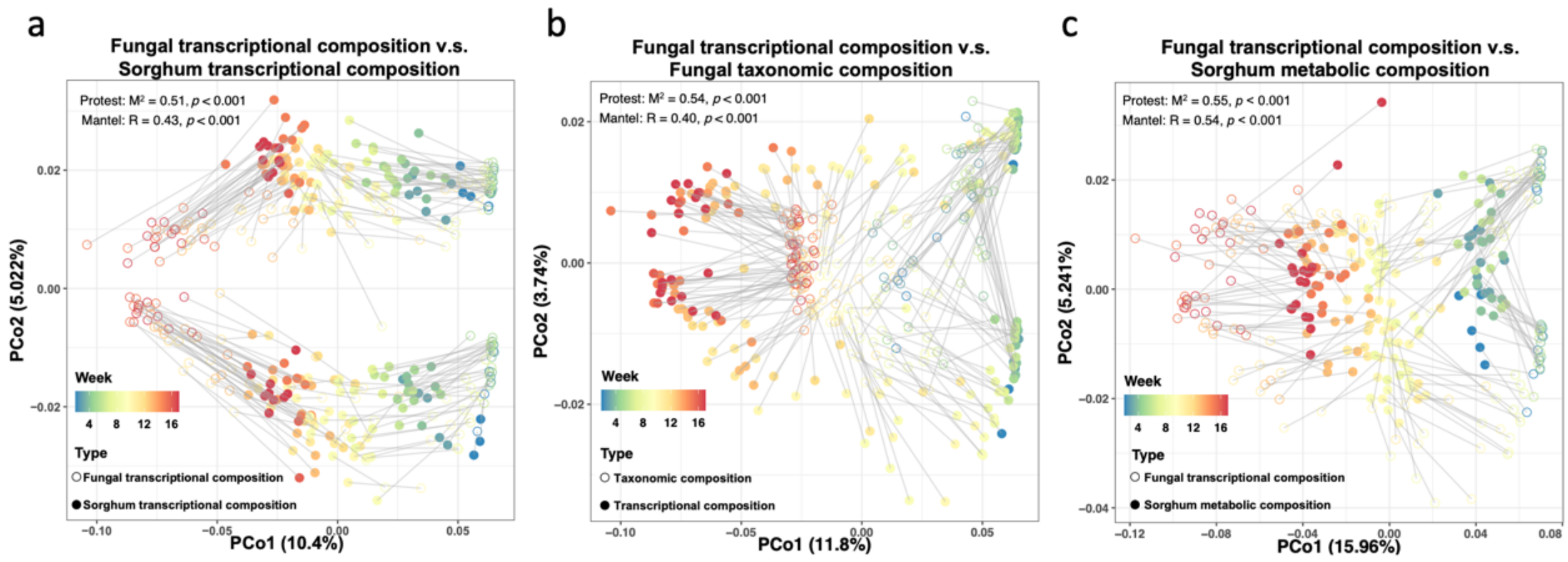

Figure S8. Associations of fungal metatranscriptomic composition with **a** sorghum transcriptomic composition (n = 196), **b** fungal taxonomic composition (n = 196), and **c** leaf metabolic composition (n = 158), as determined by Procrustes and Mantel tests. M^2 was the sum of squared pointwise differences between the two datasets, with smaller M^2 indicating a stronger correlation.

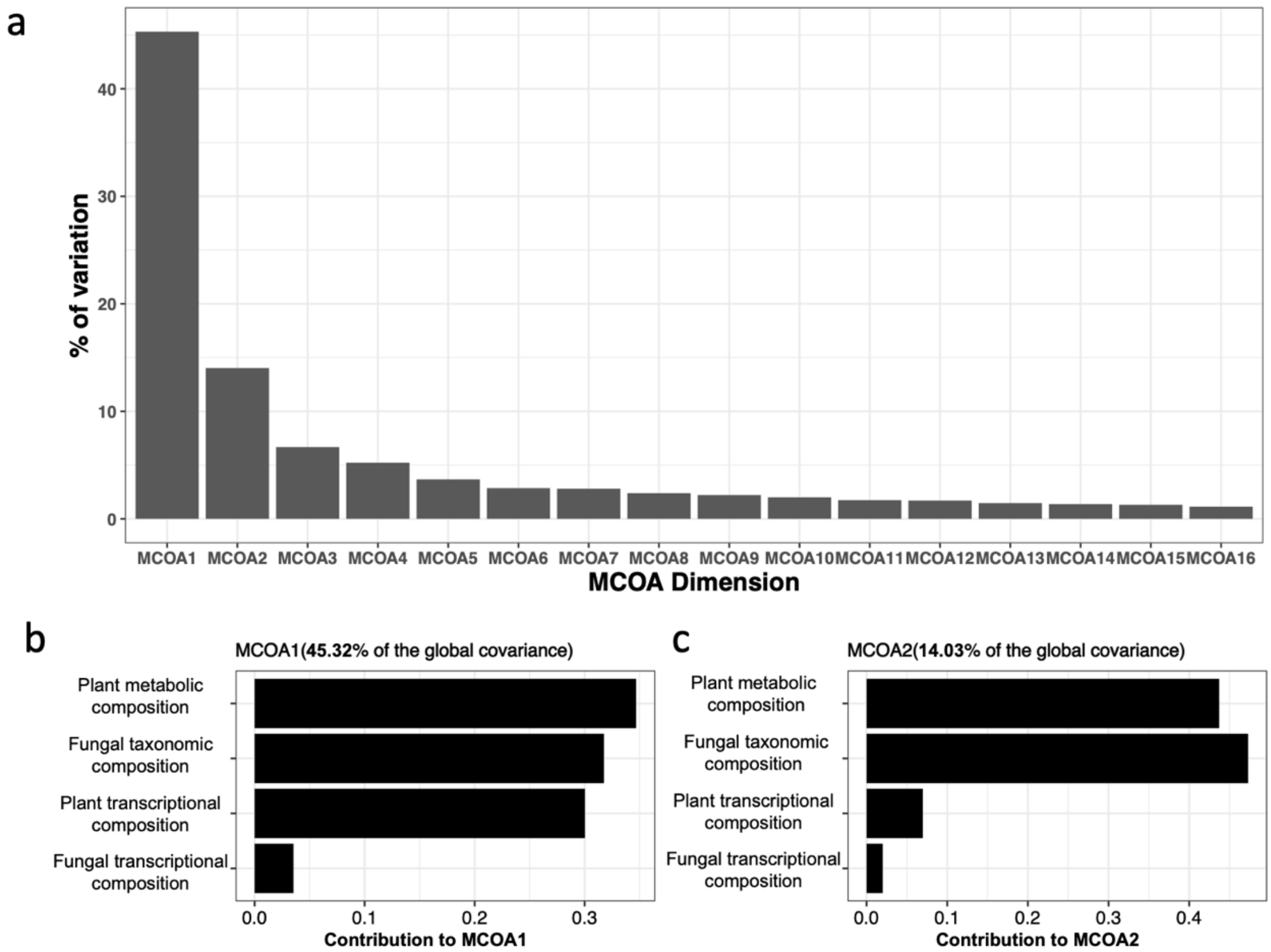

Figure S9. Multiple co-inertia analysis (MCOA) integrates holo-omics datasets. a Bar plot showed the percentage of variation in the entire dataset captured by each dimension of the MCOA. Contribution of individual datasets to **b** MCOA1 and **c** MCOA2. Analyzed datasets: fungal community composition, fungal metatranscriptomics, plant transcriptomics, and plant metabolomics.

Table S1. Genomic information for the closest reference genomes of the 25 most abundant fungal operational taxonomic units (OTUs).

OTUs	Reference genome	Genome size (Mb)	GC content (%)	Genome ID
Cladosporium OTU1	*Cladosporium cladosporioides*	33.2	52.5	GCA_002901145.1
Alternaria OTU4	*Alternaria alternata*	33.0	51.5	GCA_001642055.1
Ustilago OTU15	*Ustilago trichophora*	39.0	53.5	GCA_900323505.1
Filobasidium OTU10	*Filobasidium floriforme*	27.5	53.0	GCA_021052385.1
Setophoma OTU2	*Phoma* sp.	40.2	50.5	GCA_004835665.1
Epicoccum OTU11	*Epicoccum nigrum*	34.7	52.0	GCA_002116315.1
Sporobolomyces OTU26	*Sporobolomyces roseus*	22.4	49.5	GCA_016617785.1
Cryptococcus OTU24	*Cryptococcus* sp.	17.3	49.0	GCA_026538405.1
Symmetrospora OTU14	*Symmetrospora coprosmae*	20.2	60.0	GCA_008802785.1
Alternaria OTU27	*Alternaria ethzedia*	36.0	50.5	GCA_023757985.1
Filobasidium OTU50	*Filobasidium wieringae*	19.8	50.0	GCA_001600055.1
Alternaria OTU7	*Alternaria consortialis*	34.2	50.5	GCA_001950455.1
Aureobasidium OTU33	*Aureobasidium pullulans*	50.0	29.6	GCA_000721785.1
Acremonium OTU5	*Acremonium* sp.	35.8	52.5	GCA_021347865.1
Pseudozyma OTU88	*Moesziomyces aphidis*	17.9	61.0	GCA_000517465.1
Gibberella OTU18	*Fusarium nygamai*	51.6	47.5	GCA_001262555.1
Stemphylium OTU53	*Stemphylium vesicarium*	38.7	50.5	GCA_004380135.1
Fusarium OTU6	*Fusarium equiseti*	40.8	48.0	GCA_003313175.1
Anthracocystis OTU116	*Anthracocystis*	18.8	54.0	GCA_014826065.1
Sporisorium OTU79	*Sporisorium graminicola*	19.6	56.5	GCF_005498985.1
Fusarium OTU16	*Fusarium oxysporum*	50.4	47.5	GCF_013085055.1
Ascochyta OTU397	*Ascochyta rabiei*	40.8	49.0	GCF_004011695.2
Papiliotrema OTU61	*Papiliotrema laurentii*	19.5	56.0	GCA_031326905.1
Monosporascus OTU20	*Monosporascus cannonballus*	89.6	36.5	GCA_004154925.1

Striaticonidium OTU35	*Myrothecium inundatum*	39.2	51.0	CBS_120646

References

1 Varoquaux, N. *et al.* Transcriptomic analysis of field-droughted sorghum from seedling to maturity reveals biotic and metabolic responses. *Proc. Natl. Acad. Sci. USA* **116**, 27124–27132, doi:10.1073/pnas.1907500116 (2019).

2 Gao, C. *et al.* Successional adaptive strategies revealed by correlating arbuscular mycorrhizal fungal abundance with host plant gene expression. *Mol. Ecol.* **32**, 2674-2687, doi:10.1111/mec.16343 (2023).

3 Gao, C. *et al.* Strong succession in arbuscular mycorrhizal fungal communities. *ISME J.* **13**, 214-226, doi:10.1038/s41396-018-0264-0 (2019).
